# Prognostic and diagnostic utility of pancreatic stone protein in pediatric sepsis and mortality

**DOI:** 10.55730/1300-0144.5844

**Published:** 2024-07-07

**Authors:** Mehmet Akif DÜNDAR, Emin CERAN, Başak Nur AKYILDIZ

**Affiliations:** Division of Pediatric Critical Care Medicine, Faculty of Medicine, Erciyes University, Kayseri, Turkiye

**Keywords:** Blood culture, C-reactive protein, pediatrics, procalcitonin, pancreatic stone protein, sepsis

## Abstract

**Background/aim:**

Early detection and prognosis of sepsis in critically ill children is crucial. The aim of this research was to investigate the prognostic ability of pancreatic stone protein (PSP) in validating sepsis and predicting mortality in a prospective observational study.

**Materials and methods:**

In a single-center study, pediatric intensive care unit patients were divided into cohorts of confirmed and suspected sepsis, as well as survivors and nonsurvivors. Patients with positive blood culture growth were considered to have confirmed sepsis, while their negative counterparts were considered to have suspected sepsis. Comparisons were made between complete blood counts, laboratory parameters, mortality indices, and C-reactive protein (CRP), procalcitonin (PCT), and PSP levels. The correlations between PSP and alternative inflammatory markers and mortality indices were then analyzed. The diagnostic and prognostic applicability of PSP for sepsis confirmation and mortality prediction was assessed using receiver operating characteristic curve analysis.

**Results:**

PSP levels were significantly elevated in patients with confirmed sepsis and within the nonsurvivor segment. In confirming sepsis and predicting mortality, PSP outperformed CRP and PCT in terms of sensitivity. It had sensitivity of 95% in diagnosing sepsis at a cut-off level of 50 ng/L, with an area under the curve (AUC) of 0.67 (95% CI: 0.52–0.81), and sensitivity of 92% in predicting mortality, with an AUC of 0.71 (95% CI: 0.56–0.83). In addition, PSP showed significant correlations with CRP, PCT, and mortality scores.

**Conclusion:**

PSP is emerging as a highly sensitive marker for confirming sepsis and predicting mortality in critically ill pediatric patients. Incorporating the PSP biomarker into routine clinical practice could potentially improve the management of pediatric sepsis.

## 1. Introduction

Sepsis is a leading cause of childhood mortality worldwide, contributing to 19% of deaths and accounting for 8% of pediatric intensive care unit (PICU) admissions [1]. The identification of confirmed sepsis, typically by positive bacterial culture, requires rapid intervention to mitigate organ failure and reduce mortality [2]. Differentiating between culture-positive and culture-negative sepsis is imperative, as the Surviving Sepsis Campaign guidelines advocate starting antibiotics within the first hour of diagnosis [3]. Delaying the start of antibiotics correlates with a 7.6% increase in mortality from septic shock. In cases of culture-negative sepsis, unwarranted use of antibiotics can lead to antibiotic resistance [4]. A key component of meticulous care is patient stratification, which is achieved by assessing the risk of mortality and the potential benefit of early aggressive treatment [5].

Biomarkers have the ability to significantly influence the confirmation of sepsis and indicate its severity [6]. However, the exact role of biomarkers in the management of patients with sepsis remains unclear and further research is needed to identify specific biomarkers attributable to sepsis. A strikingly increased serum concentration of pancreatic stone protein (PSP) is observed in cases of sepsis [7]. PSP, a protein belonging to the C-type lectins, is essential for the activation of polymorphonuclear cells and has proinflammatory activity in vitro. Several studies have described an increase in PSP levels that precedes the manifestation of sepsis and persists throughout the progression to septic shock [8]. However, only a handful of studies suggest that PSP may prove to be an effective biomarker for the early diagnosis and prognosis of sepsis in the pediatric population [9].

Currently, there is a lack of definitive markers for blood culture growth in critically ill children to confirm sepsis and predict outcomes. This study aims to evaluate the prognostic ability of PSP compared to other inflammatory markers, with a focus on its efficacy in both confirming sepsis and predicting survival in critically ill pediatric patients with sepsis.

## 2. Materials and methods

In this prospective study, 48 patients aged between 1 month and 18 years of age were admitted to the PICU of Erciyes University between January and April 2022 with suspected sepsis. Ethics approval was obtained from the institutional ethics committee with decision number 2022/30, and informed consent was obtained from all participants or their families. Blood samples were collected from patients presenting with symptoms of systemic inflammatory response syndrome (SIRS) including fever or hypothermia, as defined by the 2021 International Pediatric Sepsis Guidelines. However, no patients in our study developed hypothermia. Those with suspected sepsis underwent a comprehensive set of tests including complete blood count, blood culture, and measurements of glucose, blood urea nitrogen (BUN), creatinine, aspartate aminotransferase (AST), alanine aminotransferase (ALT), albumin, and the inflammatory markers of C-reactive protein (CRP), procalcitonin (PCT), and PSP.

### 2.1. Patient grouping and criteria

Two separate assessment scenarios were used. The first compared patients with suspected sepsis (n = 28, 58.3%) to those with confirmed sepsis (n = 20, 41.7%). The second differentiated between survivors (n = 34, 70.8%) and nonsurvivors (n = 14, 29.2%). Patients with SIRS symptoms and fever whose blood cultures showed bacterial growth were categorized within the confirmed sepsis group. Those with negative blood cultures constituted the suspected sepsis group. Patients with functional disorders unrelated to sepsis and those who had undergone surgery or had renal, hepatic, or cardiac failure were excluded from the study.

### 2.2. Parameters and scores

Scores for the Pediatric Risk of Mortality (PRISM II), Pediatric Logistic Organ Dysfunction (PELOD-2), and Pediatric Sequential Organ Failure Assessment (pSOFA) were recorded for each patient. Other variables such as underlying diseases, age, sex, type of oxygen therapy, length of hospital stay, and presence of a central venous catheter were also recorded. Laboratory and blood count parameters, inflammatory markers, and mortality rates were then compared between the groups. Further analyses aimed to determine the prognostic value of CRP, PCT, and PSP in confirming sepsis and predicting mortality.

### 2.3. PSP measurement procedure

An isoform-specific enzyme-linked immunosorbent assay (ELISA) was used to assess PSP at the bedside. Fresh venous or capillary blood was transferred to a tube containing K^3^-EDTA using a 30-μL micropipette. This blood was then transferred to Abiomix reagent (Abiomix, Dubai, UAE). After opening the Abiomix bottle, the contents were extracted with a 30-μL micropipette and dispensed into the white part of a capsule. The capsule was sealed and the device was placed in an ABIONIC machine (ABIONIC, Lausanne, Switzerland). Results were obtained in approximately 5 min.

### 2.4. Statistical analysis

**Sample size was determined by power analysis based on the study by Wu et al. [10]. Calculations were perfo**rmed using G*Power 3.1.9.2 software, resulting in 36 patients total (18 for each group) with 90% power (beta = 0.1), a type-1 error rate of 5% (alpha = 0.05), and effect size of 1.143. The power analysis was based on survivor and nonsurvivor groups. Data were analyzed using IBM SPSS Statistics 22.0 (IBM Corp., Armonk, NY, USA). Histograms, q-q plots, and the Shapiro–Wilk test were used to assess data distribution. For parameters showing normal distribution, the independent sample t-test was used to compare the two groups and the results were expressed as mean ± standard deviation. For parameters not showing normal distribution, the Mann–Whitney U test was used and the results were expressed as median (25th–75th percentiles). The chi-square test was used for categorical data, with Fisher exact correction applied when necessary. Spearman correlation analysis was used to examine the association between PSP and markers of inflammation and mortality. The Youden method and receiver operating characteristic (ROC) analysis were used to assess the predictive value of the PCT, CRP and PSP markers. Values of p < 0.05 were considered statistically significant.

## 3. Results

### 3.1. Demographics of groups compared for sepsis and survival

Table 1 shows the demographics of the 48 pediatric patients admitted to the PICU and compared for sepsis and survival. There were 28 patients in the suspected sepsis group and 20 in the confirmed sepsis group. In terms of survival, there were 34 survivors and 14 nonsurvivors. The sepsis groups were similar with respect to age, weight, sex, intubation, presence of a central venous catheter, and length of stay in the PICU (p = 0.92, 0.35, 0.56, 0.23, 0.37, and 0.45, respectively). In the confirmed sepsis group, 8 patients had gram-positive infections (40%), 10 had gram-negative infections (50%), and 2 had fungal infections (10%). There were no significant differences between survivors and nonsurvivors in terms of age, weight, sex, and intubation (p = 0.75, 0.36, 0.93, 0.07, and 0.09). However, length of stay in the PICU was significantly longer among nonsurvivors (40 days; range: 8–83) than survivors (10 days; range: 3–26) (p = 0.01). In the nonsurvivor group, 7 patients had gram-negative infections (50%) and 1 patient had a fungal infection (7%). In the survivor group, 8 patients (23%) had gram-positive infections, 3 (9%) had gram-negative infections, and 1 (3%) had a fungal infection. The difference in blood culture growths between the groups was statistically significant (p = 0.01).

### 3.2. Comparisons between confirmed and suspected sepsis groups for complete blood counts, biochemical parameters, inflammatory markers, and scoring systems

No significant difference was found between the confirmed and suspected sepsis groups with regard to hemoglobin, white blood cell count, platelet count, BUN, creatinine, AST, ALT, or albumin (Table 2). The absolute neutrophil count was significantly higher in the confirmed sepsis group (11,546 ± 7827/mm^3^) compared to the suspected sepsis group (7950 ± 6294/mm^3^) (p = 0.05). No statistically significant difference was found between the groups in terms of PRISM II, PELOD-2, or pSOFA scores (Table 2). PSP, CRP, and PCT levels were significantly higher in the confirmed sepsis group (126.5 (range: 70–271) ng/L, 132 (range: 80.6–194.5) mg/dL, and 13.8 (range: 5.2–32.1) mg/dL) compared to the suspected sepsis group (87 (32–138.5) ng/L, 70.25 (43–122) mg/dL, and 7.25 (0.7–14.6) mg/dL) (p = 0.04, 0.01, and 0.05, respectively; Table 2).

### 3.3. Comparison of complete blood counts, biochemical parameters, inflammatory markers, and scoring systems between survivor and nonsurvivor groups

No statistically significant difference was found between the survivor and nonsurvivor groups for white blood cell count, hemoglobin, platelet count, absolute neutrophil count, BUN, AST, ALT, or albumin (Table 3). Creatinine levels were significantly higher in the nonsurvivor group (0.63 ± 0.24 mg/dL) compared to the survivor group (0.44 ± 0.27 mg/dL) (p = 0.03). PRISM II, PELOD-2, and pSOFA scores were significantly higher in nonsurvivors (26.5 (range: 24–30.75), 26 (range: 22–31), and 12.5 (8–18.5)) than in survivors (10.5 (range: 3.75–16), 9.5 (range: 2–12), and 4 (2–8)) (p < 0.001, p = 0.002, and p < 0.001 respectively; Table 3). PCT levels were not significantly different between the groups, whereas PSP and CRP levels were significantly higher in the nonsurvivor group (161.5 (range: 83.5–260.5) ng/L) and 129 (range: 102.5–215.5) mg/dL)) than in the survivor group (79.5 (range: 45.5–162.5 ng/L) and 70.9 (range: 41.8–132 mg/dL)) (p = 0.01 for both; Table 3).

### 3.4. Correlations of PSP with other inflammatory markers and mortality scores

PSP was positively, moderately, and statistically significantly correlated with CRP (r = 0.52, p < 0.001), PCT (r = 0.49, p < 0.001), and PRISM II (r = 0.41, p = 0.006), PELOD-2 (r = 0.44, p = 0.001), and pSOFA (r = 0.50, p < 0.001) scores (Table 4). PSP was not significantly correlated with any other laboratory parameters.

### 3.5. Comparison of the prognostic value of CRP, PCT, and PSP by ROC analysis in confirming sepsis and predicting mortality

**Figure 1:**
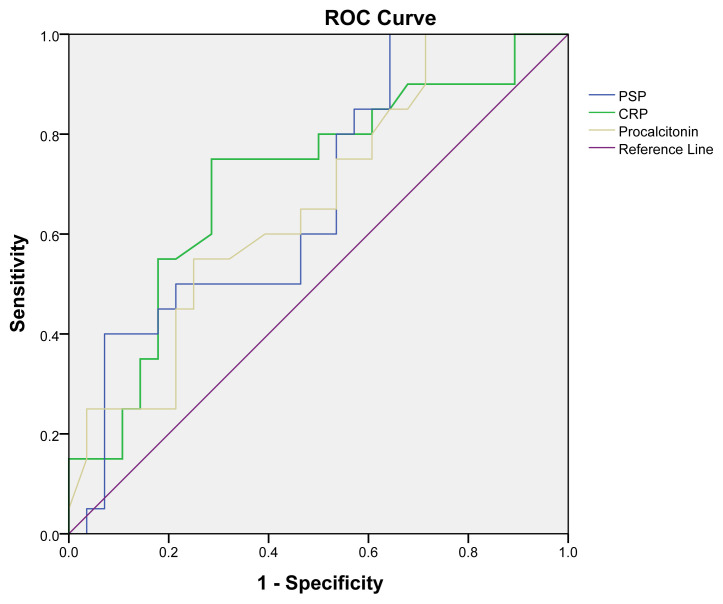
ROC curves of pancreatic stone protein (PSP), C-reactive protein (CRP), and procalcitonin in cases of confirmed sepsis.

PSP, CRP, and PCT were statistically significant in confirming sepsis (p = 0.002, 0.01, and 0.03, respectively). The area under the curve (AUC) for CRP was 0.70 (0.55–0.85), the AUC for PCT was 0.66 (0.51–0.81), and the AUC for PSP was 0.67 (0.52–0.81) with 95% confidence intervals (CIs). The sensitivity for confirming sepsis was found to be 95% for PSP, 75% for CRP, and 55% for PCT. Specificity values were 45% for PSP, 71% for CRP, and 31% for PCT. PSP of ≥50 ng/L, PCT of ≥12.5 mg/dL, and CRP of ≥155 mg/dL were used as cut-off values to confirm sepsis (Table 5; Figure 1).

**Figure 2:**
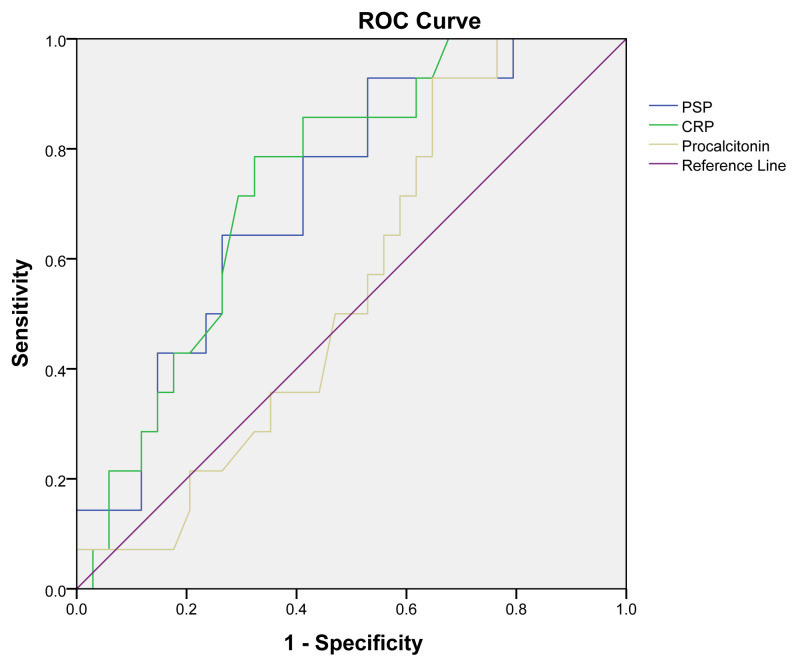
ROC curves of pancreatic stone protein (PSP), C-reactive protein (CRP), and procalcitonin in nonsurvivors.

PSP and CRP were statistically significant in predicting membership in the nonsurvivor group, whereas PCT was not (p = 0.006, 0.001, and 0.56, respectively). The AUC for PSP was 0.71 (95% CI: 0.56–0.83). The AUC for CRP was 0.73 (95% CI: 0.59–0.85). Sensitivity was 92% (0.68–0.98) for PSP and 78% (0.52–0.92) for CRP. Specificity was 47% (0.31–0.63) for PSP and 67% (0.50–0.80) for CRP (Table 5; Figure 2)

## 4. Discussion

The primary objective of this study was to evaluate the prognostic ability of PSP in comparison to other inflammatory markers, with a particular focus on the confirmation of sepsis and the prediction of mortality in critically ill pediatric patients. PSP not only demonstrated remarkable sensitivity in these areas but also showed correlations with other inflammatory markers and mortality outcomes, thus positioning itself as a potentially vital biomarker for both confirmation and prognosis of sepsis in children.

PSP has demonstrated its sensitivity in validating blood culture growth and predicting mortality in critically ill children hospitalized for suspected sepsis. We observed significantly elevated serum levels of PSP, CRP, and PCT in patients with confirmed sepsis compared to those with suspected sepsis. The AUC values for these biomarkers underscore their importance as key acute-phase reactants in sepsis confirmation and highlight the critical nature of accurate sepsis validation and mortality prediction in pediatric ICUs. For example, a recent study identified elevated PSP and CRP levels in patients with sepsis, with PSP predicting pediatric sepsis with 80% sensitivity and AUC values for CRP and PSP of 0.917 and 0.868, respectively [11].

At the same time, other studies have shown elevated PSP levels in infected infants, with AUC values of 0.69 for PSP, 0.77 for PCT, and 0.66 for CRP [12]. Such findings are consistent with other previous studies validating the efficacy of PSP in identifying infections and differentiating sepsis patients in the emergency department [13]. In addition, one study highlighted elevated PSP levels in infected neonates compared to noninfected neonates [14].

In our cohort, serum levels of PSP and CRP were significantly higher among nonsurvivors compared to survivors. With its outstanding sensitivity for confirming sepsis and predicting mortality, PSP may play a pivotal role in the early detection and management of sepsis in critically ill pediatric populations. Several studies, including ours, have highlighted the potential of serum PCT, CRP, and PSP levels as key biomarkers for risk stratification in pediatric sepsis [12]. Huo et al. highlighted a significant inverse relationship between serum PCT, CRP, and PSP levels and the Pediatric Critical Illness Score, strengthening their prognostic value [15].

Our results echo the findings of Saleh et al., who suggested that PSP and pSOFA emerged as the most important discriminators of mortality, with PSP detecting pediatric sepsis with high sensitivity and both PSP and pSOFA having substantial prognostic value for predicting mortality, with AUCs of 0.709 [11]. In addition, another study found that PSP levels did not fluctuate significantly between patients with systemic inflammatory response syndrome and sepsis until signs of organ dysfunction became apparent, suggesting that patients with heightened PSP levels often had more severe outcomes, especially those with high PELOD scores or multiple organ failure [16].

However, it is important to acknowledge the limitations of our study. The relatively small patient sample size and its single-center nature may potentially limit its broader applicability, and further multicenter studies with different patient demographics are needed to validate our findings.

In conclusion, this study highlights the importance of early detection and skilled management of sepsis in the PICU. PSP is emerging as a promising biomarker with commendable diagnostic and prognostic capabilities. Incorporating PSP into sepsis management protocols may pave the way for more refined therapeutic strategies and improved patient outcomes. Subsequent research should explore the collective efficacy of PSP with other inflammatory markers to further improve diagnostic and prognostic accuracy in pediatric sepsis. The integration of biomarkers such as PSP into routine clinical practice may enable healthcare professionals to make more informed decisions, potentially raising the standard of care in pediatric sepsis.

## Figures and Tables

**Table 1:** Demographic data of the groups compared in terms of sepsis and mortality.

Confirmed sepsis (n = 20)	Suspected sepsis (n = 28)	p	Parameters	Survivors (n = 34)	Nonsurvivors (n = 14)	p
74.6 ± 65.7	68.8 ± 58	0.92	Age, months	71.2 ± 60.9	71.4 ± 63.3	0.75
17.3 ± 16.1	21.2 ± 18.2	0.35	Weight, kg	20.7 ± 17.3	16.9 ± 17.6	0.36
10/10 (50%/50%)	11/17 (39%/61%)	0.56	Female/male, n (%)	15/19 (44%/56%)	6/8 (43%/57%)	0.93
17/20 (85%)	21/28 (75%)	0.23	Intubation, n (%)	24/34 (70%)	14/14 (100%)	0.07
19/20 (95%)	23/28 (82%)	0.37	Central venous catheter, n (%)	28/34 (82)	14/14 (100%)	0.09
16.5 (5–69)	11 (4–28)	0.45	PICU stay, days	10 (3–26)	40 (8–83)	0.01
Gram+: 8, 40% Gram–: 10, 50% Fungus: 2, 10%	-	-	Blood culture growth microorganism	Gram+: 8, 23% Gram–: 3, 9% Fungus: 1, 3%	Gram+: 0 Gram–: 7, 50% Fungus: 1, 7%	0.01

**Table 2:** Comparisons between confirmed and suspected sepsis groups for complete blood counts, biochemical parameters, inflammatory markers, and scoring systems.

Parameters	Confirmed sepsis (n = 20)	Suspected sepsis (n = 28)	p
**Laboratory parameters**			
Hemoglobin, g/dL	10.10 ± 1.7	9.7 ± 1.8	0.47
White blood cells, mm^3^	15,684 ± 10,429	11,453 ± 7480	0.08
Absolute neutrophils, mm^3^	11,546 ± 7827	7950 ± 6294	0.05
Platelets, mm^3^/10^3^	221 ± 228	226 ± 185	0.58
BUN, mg/dL	18.8 ± 12.3	16.2 ± 6.1	0.92
Creatine, mg/dL	0.49 ± 0.29	0.50 ± 0.26	0.84
AST, U/L	75 ± 79	68 ± 74	0.57
ALT, U/L	48 ± 80	45 ± 58	0.47
Albumin, g/dL	2.76 ± 0.75	2.82 ± 0.63	0.63
**Mortality scores**			
PRISM II score	14 (8.25–26)	11.5 (3.75–25.5)	0.52
PELOD-2 score	11.5 (9.25–27)	11 (2–26.5)	0.59
pSOFA score	7 (3–12)	5.5 (2–9)	0.27
**Inflammatory markers**			
PSP, ng/L	126.5 (70–271)	87 (32–138.5)	0.04
CRP, mg/dL	132 (80.6–194.5)	70.25 (43–122)	0.01
PCT, mg/dL	13.8 (5.2–32.1)	7.25 (0.7–14.6)	0.05

**Table 3:** Comparison of complete blood counts, biochemistry, inflammatory markers, and scoring systems between survivor and nonsurvivor groups.

Parameters	Survivors (n = 34)	Nonsurvivors (n = 14)	p
**Laboratory parameters**			
Hemoglobin, g/dL	10.09 ± 1.87	9.48 ± 1.61	0.26
White blood cells, mm^3^	12,093 ± 8262	15,942 ± 10,329	0.14
Absolute neutrophils, mm^3^	8279 ± 6323	12,623 ± 8393	0.06
Platelets, mm^3^/10^3^	238 ± 221	189 ± 146	0.54
BUN, mg/dL	16.3 ± 7.7	19.5 ± 12.1	0.57
Creatine, mg/dL	0.44 ± 0.27	0.63 ± 0.24	0.03
AST, U/L	72 ± 84	69 ± 53	0.85
ALT, U/L	51 ± 75	34 ± 44	0.33
Albumin, g/dL	2.89 ± 0.68	2.57 ± 0.63	0.24
**Mortality scores**			
PRISM II score	10.5 (3.75–16)	26.5 (24–30.75)	<0.001
PELOD-2 score	9.5 (2–12)	26 (22–31)	0.002
pSOFA score	4 (2–8)	12.5 (8–18.5)	<0.001
**Inflammatory markers**			
PSP, ng/L	79.5 (45.5–162.5)	161.5 (83.5–260.5)	0.01
CRP, mg/dL	70.9 (41.8–132)	129 (102.5–215.5)	0.01
PCT, mg/dL	8.3 (2.5–18.2)	8.5 (4.9–19.7)	0.59

**Table 4:** Correlations of PSP with other inflammatory markers and mortality scores.

Variable	Correlation (r)	p
PSP versus CRP	0.52	<0.001
PSP versus PCT	0.49	<0.001
PSP versus PRISM II	0.41	0.006
PSP versus PELOD-2	0.44	0.001
PSP versus pSOFA	0.50	<0.001

**Table 5:** Comparison of the prognostic values of CRP, PCT, and PSP by ROC analysis in the confirmation of sepsis and prediction of mortality.

	Values	AUC	95% CI	p	Cut-off	Sensitivity	Specificity
**Confirmed sepsis Group**	PSP	0.67	0.52–0.81	0.02	>50	95% (0.83–1.0)	45% (0.20–0.54)
	CRP	0.70	0.55–0.85	0.001	>110	75% (0.53–0.88)	71% (0.52–0.84)
	PCT	0.66	0.51–0.81	0.03	>12.5	55% (0.34–0.74)	35% (0.56–0.87)
**Nonsurvivors Group**	PSP	0.71	0.56–0.83	0.006	>70	92% (0.68–0.98)	47% (0.31–0.63)
	CRP	0.73	0.59–0.85	0.001	>111	78% (0.52–0.92)	67% (0.50–0.80)
	PCT	0.54	0.39–0.69	0.56	-	-	-
